# A clinically relevant computed tomography (CT) radiomics strategy for intracranial rodent brain tumour monitoring

**DOI:** 10.1038/s41598-024-52960-1

**Published:** 2024-02-01

**Authors:** Kate Connor, Emer Conroy, Kieron White, Liam P. Shiels, Simon Keek, Abdalla Ibrahim, William M. Gallagher, Kieron J. Sweeney, James Clerkin, David O’Brien, Jane B. Cryan, Philip J. O’Halloran, Josephine Heffernan, Francesca Brett, Philippe Lambin, Henry C. Woodruff, Annette T. Byrne

**Affiliations:** 1 Department of Physiology and Medical Physics, Royal College of Surgeons in Ireland, York Street, Dublin 2, Ireland; 2 National Pre-Clinical Imaging Centre (NPIC), Dublin, Ireland; 3 UCD School of Biomolecular and Biomedical Science, UCD Conway Institute, University College Dublin, Belfield, Dublin, Ireland; 4 The D-Lab: Department of Precision Medicine, GROW - School for Oncology and Reproduction, Maastricht University, Maastricht, The Netherlands; 5 Department of Neurosurgery, Beaumont Hospital, Dublin, Ireland; 6 Department of Neurosurgery, Queen Elizabeth Hospital, Birmingham, UK; 7 Department of Neuropathology, Beaumont Hospital, Dublin, Ireland; 8 Department of Radiology and Nuclear Medicine, GROW - School for Oncology and Reproduction, Maastricht University Medical Centre+, Maastricht, The Netherlands

**Keywords:** Cancer imaging, Cancer, Animal disease models, X-ray tomography

## Abstract

Here, we establish a CT-radiomics based method for application in invasive, orthotopic rodent brain tumour models. Twenty four NOD/SCID mice were implanted with U87R-Luc2 GBM cells and longitudinally imaged via contrast enhanced (CE-CT) imaging. Pyradiomics was employed to extract CT-radiomic features from the tumour-implanted hemisphere and non-tumour-implanted hemisphere of acquired CT-scans. Inter-correlated features were removed (Spearman correlation > 0.85) and remaining features underwent predictive analysis (recursive feature elimination or Boruta algorithm). An area under the curve of the receiver operating characteristic curve was implemented to evaluate radiomic features for their capacity to predict defined outcomes. Firstly, we identified a subset of radiomic features which distinguish the tumour-implanted hemisphere and non- tumour-implanted hemisphere (i.e, tumour presence from normal tissue). Secondly, we successfully translate preclinical CT-radiomic pipelines to GBM patient CT scans (*n* = 10), identifying similar trends in tumour-specific feature intensities (E.g. ‘glszm Zone Entropy’), thereby suggesting a mouse-to-human species conservation (a conservation of radiomic features across species). Thirdly, comparison of features across timepoints identify features which support preclinical tumour detection earlier than is possible by visual assessment of CT scans. This work establishes robust, preclinical CT-radiomic pipelines and describes the application of CE-CT for in-depth orthotopic brain tumour monitoring. Overall we provide evidence for the role of pre-clinical ‘discovery’ radiomics in the neuro-oncology space.

## Introduction

Glioblastoma (GBM) is an invasive, heterogeneous, and incurable malignancy^1,2^ with patients demonstrating a low median life expectancy (< 2 years). Radiological imaging is an integral aspect of disease management. Indeed, non-invasive monitoring methods are essential in the neuro-oncology setting, due to the intrinsic difficulties associated with intracranial biopsy^3^. In this context, Magnetic Resonance Imaging (MRI) and Computed Tomography (CT) underpin diagnosis^4^, longitudinal disease management, and monitoring of therapeutic response^5^. Nevertheless, while MRI is considered the ‘standard of care’ imaging modality in the clinic, the diagnostic value of CT imaging remains pertinent^6,7^. Specifically, patients with suspected intracranial malignancies undergo CT at first diagnosis, during radiotherapy treatment planning, and when MRI is precluded (e.g. due to the presence of pacemaker, or older aneurysm clips)^6^.Response Assessment in Neuro-Oncology (RANO) criteria which are implemented in the assessment of patient CT and MRI scans^8,9^ facilitate meaningful disease surveillance, evaluation of radiographic response and progression^9^, and represent the gold standard for first-line treatment response evaluation in GBM.

Radiomic analysis of medical images is a disruptive methodology which allows the extraction of quantitative features from radiological scans. Overall, radiomics has emerged as a powerful method to gain clinically relevant insights into tumour biology, patient prognosis and treatment prediction. In the context of brain tumours, radiomic feature analysis may enhance the use of the RANO criteria (as highlighted by the emergence of the ‘Artificial Intelligence and Imaging Response (AI-RANO) Criteria’^10^). Moreover, radiomics^11,12^ has been shown to support clinical assessment of glioma staging^13,14^, characterisation of GBM tumour heterogeneity^15^ and may reveal critical information underpinning tumour phenotype^16,17^, response and progression. Most recently, radiomic analysis which has largely focused on MR-based radiomic feature assessment, has been ‘reverse translated’ to pre-clinical scan data^18^.

**Figure 1:**
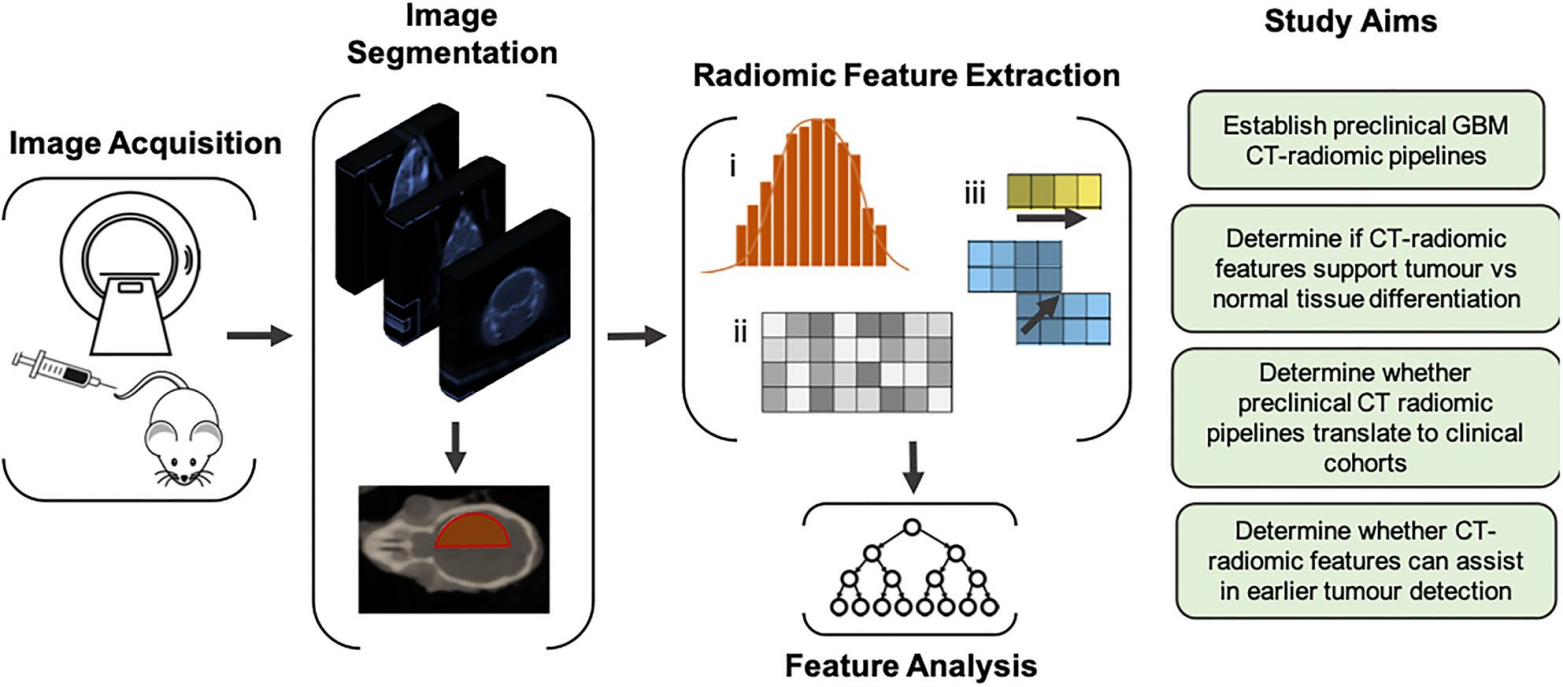
*Graphical representation of experimental workflow and aims.* The Invasive U87R-Luc2 orthotopic murine GBM model was firstly established. Longitudinal CE-CT imaging was performed using a dedicated small animal CT-system. Following image segmentation, radiomic analysis, including histogram (1st order), grey level cooccurrence matrix (GLCM) or second order and higher order features, was performed to assess alterations over time and between normal and tumour tissue. Recursive Feature Elimination or Boruta algorithm were implemented as feature selection algorithms.

In the present study we sought to establish a CT-radiomics pipeline for application in the monitoring of pre-clinical GBM models. Specifically, we demonstrate the utility of pre-clinical CT-radiomics to distinguish image features and characteristics which are not visible by eye. Importantly, we show that CT-radiomic features facilitate differentiation of the tumour implanted hemisphere when compared to the non-tumour implanted hemisphere on CE-CT scans. Additionally, we show that, rodent-derived CT-features interrogated in a clinical dataset manifest analogous trends in feature distribution (i.e. similarly increased intensity), suggesting a mouse-to-human species conservation (a conservation of radiomic features across species^18^). Moreover, our hypothesis generating study indicates that radiomic features may assist in the identification of tumours by CE-CT earlier than is possible by visual assessment of pre-clinical CT images (Fig. 1).

## Results

### CT-radiomic workflow for analysis of orthotopic GBM tumours

**Figure 2:**
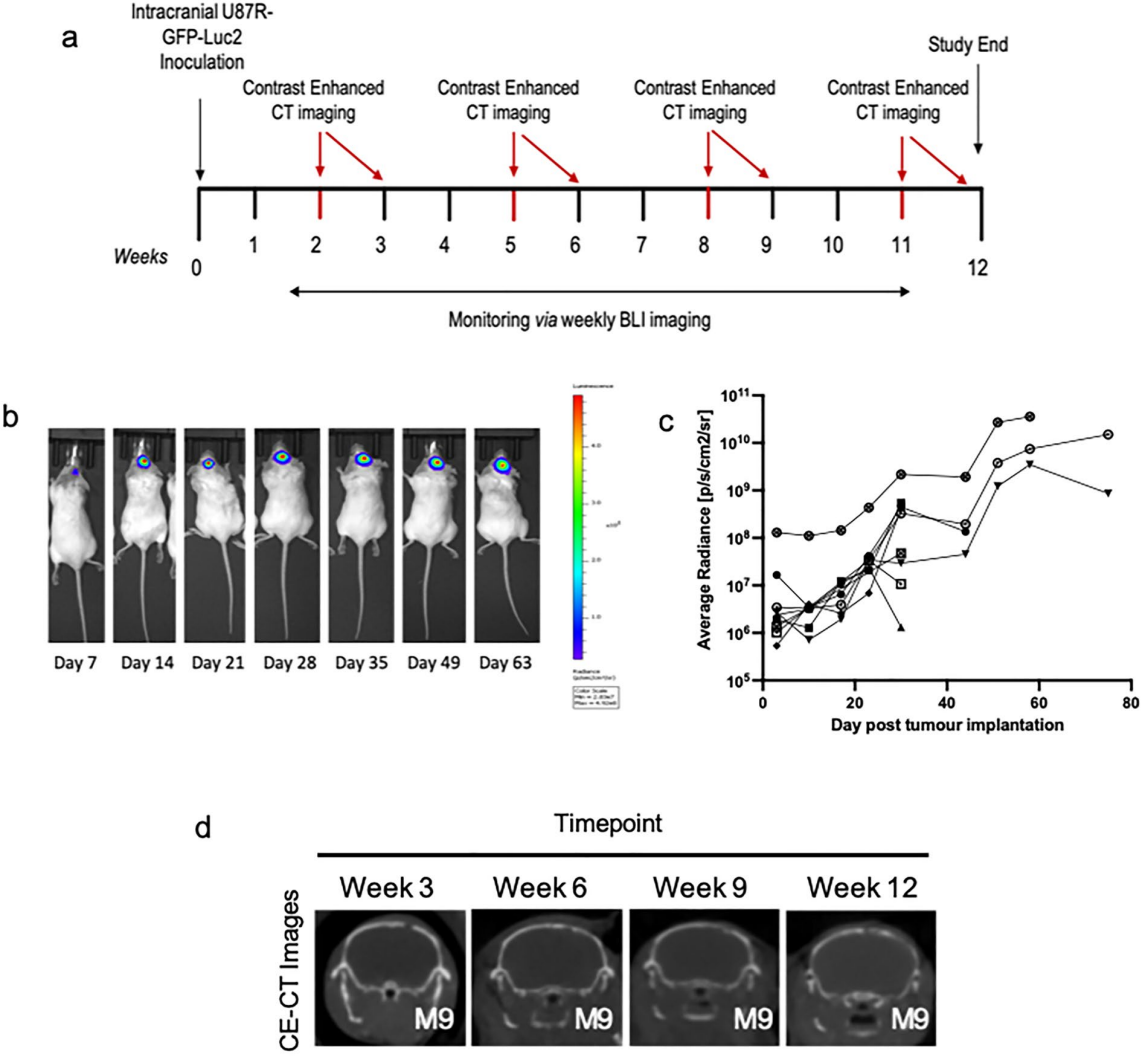
*Overview of In vivo study design and imaging data collected. *(**a**) Schematic of U87R-Luc2 model imaging schedule. (**b**) Representative BL image of U87R-Luc2 tumour bearing NOD/SCID mouse. (**c**) Bioluminescence data showing U87R-Luc2 tumour growth in each animal as average radiance [p/s/cm2/sr]. (**d**) Representative reoriented CE-CT images of orthotopically growing U87R-Luc2 tumour bearing mice imaged over 4 timepoints (Week-3,- 6, -9 and -12; coronal plane).

To begin, we performed longitudinal CE-CT imaging of U87R-Luc2 orthotopic tumour bearing NOD/SCID mice (n = 10, Fig. S1a) (Figs. 2a, S1c). Tumour-bearing mice underwent CE-CT imaging (Figs. 2d, S1c) at three-weekly intervals. Mice were monitored until reaching humane endpoint (Fig. S1a). Upon visual inspection (blinded/randomized), 16% of CE-CT images were identified as positive for tumour presence (Fig. S1d). Throughout, tumour growth was monitored via weekly BLI (Fig. 2b) and tumour presence was confirmed post mortem via immunohistochemistry (IHC). IHC analysis further confirmed a consistent phenotype across all animals with no significant difference in proliferation (Ki67) or vessel density (CD31). (Fig. S1b). In all, tumours were detectable using BLI one week (Week-1) following tumour implantation (Fig. 2c). In contrast, tumours were only detectable upon visual inspection in 16% of CE-CT images overall, with none detectable by eye before week 6 (Fig. S1d). As expected, these data confirm that BLI is a more sensitive method for pre-clinical intracranial tumour detection than CE-CT imaging^19–22^. Interestingly, mouse 9 (M9) displays both a strong BLI signal at day 75 (Fig. 2c) and an extended survival time compared to other animals (76 days *vs* 50.5 days median survival; Supplementary Figure S1a). Indeed, visual inspection of M9 at the time of euthanasia highlighted the presence of a tumour located in close proximity to the skull, thus underpinning this antipodal relationship between BLI signal and survival time (Fig. 2c; anecdotal observation).

**Figure 3:**
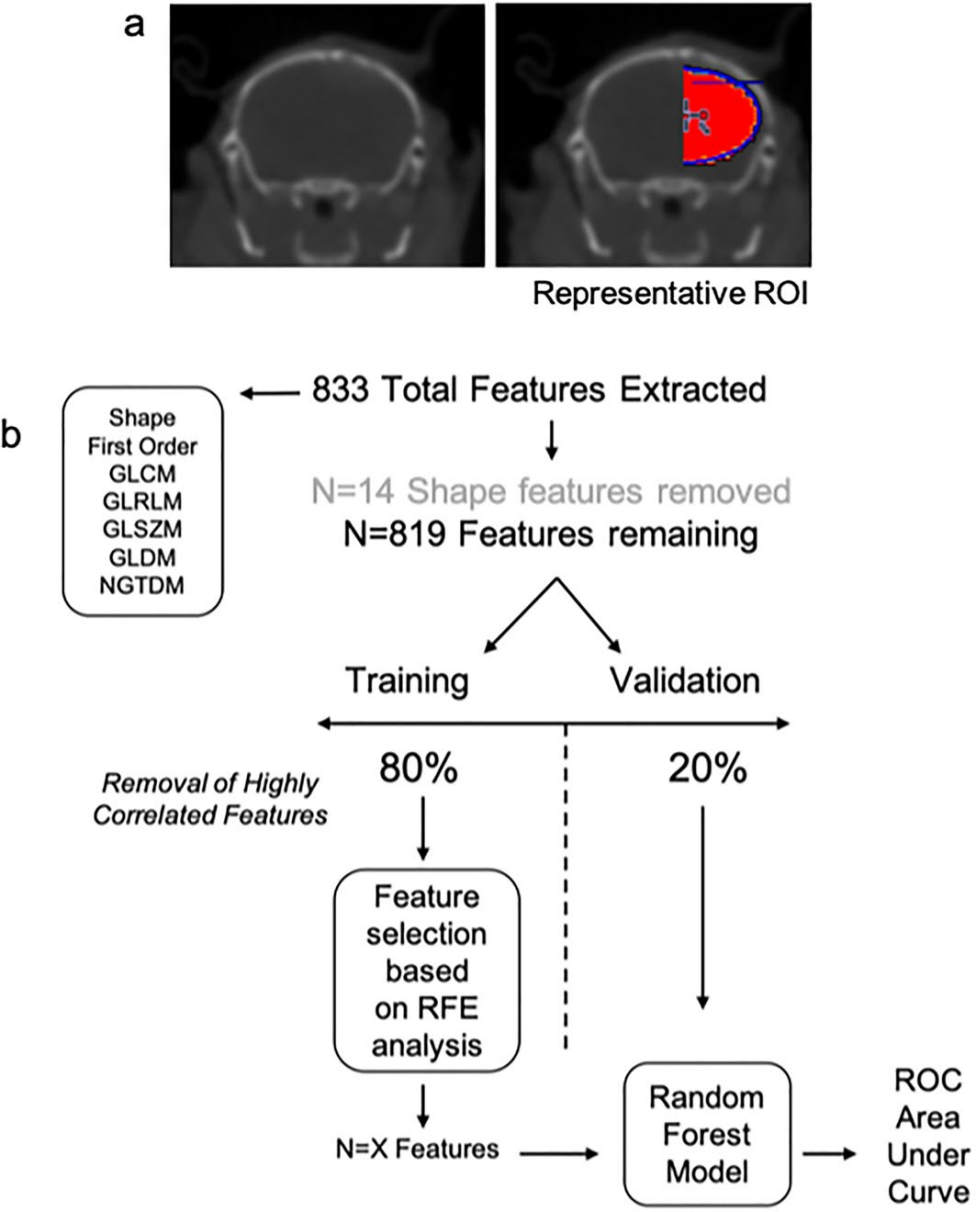
*Preclinical radiomic pipeline. *Representative axial images of segmentations regions (red). (**a**) Semi-automatic delineation of CT images in DICOM format was performed using PMOD (version 3.9). 3D Volume-of-interests (VOIs) were delineated for both right hemisphere (RH; tumour-implanted hemisphere) and the left hemisphere (LH; non-tumour-implanted hemisphere). (**b**) Radiomic features (*n* = 833) were partitioned into training and validation datasets (80:20). Remaining uncorrelated feature number following pair-wise Spearman correlation, and AUC and ROC results are determined for each comparison.

Next, preclinical GBM radiomic feature extraction pipelines were established (Figs. 1, 3). Delineation of reconstructed CE-CT images in DICOM format was performed using PMOD software (version 3.9; PMOD Technologies). A threshold was determined per image to exclude bone from generated bounding boxes (Fig. 3a). 3D Volume of interests (VOIs) were delineated for both the right hemisphere (RH; tumour-implanted hemisphere, whole hemisphere delineated) and left hemisphere (LH; non-tumour-implanted hemisphere, whole hemisphere delineated) of each animal. ROI binary masks were generated and stored in a 3D volume NIfTI format. Feature extraction via PyRadiomics successfully extracted a total of 833 features for both LH and RH (Fig. 3b) including: shape (n = 14), first order statistics (n = 18), gray level cooccurrence matrix (GLCM) (n = 22), gray level run length matrix (GLRLM) (n = 16), gray level size zone matrix (GLSZM) (n = 16), gray level dependence matrix (GLDM) (n = 14) and neighbouring gray tone difference matrix (NGTDM) (n = 5). Wavelet filtering was subsequently applied to these features. Shape features were excluded from all analyses (Fig. S2). Definitions for radiomics features are available online within the Pyradiomics documentation (https://pyradiomics.readthedocs.io/en/latest/features.html). Notably, while inclusion of wavelet filtering of images increases data dimensionality, recent studies have demonstrated that inclusion of wavelet features increases the reproducibility and repeatability of radiomic signatures^23^.

### Selection of CT radiomic features which discriminate tumour *vs* normal tissue

**Figure 4:**
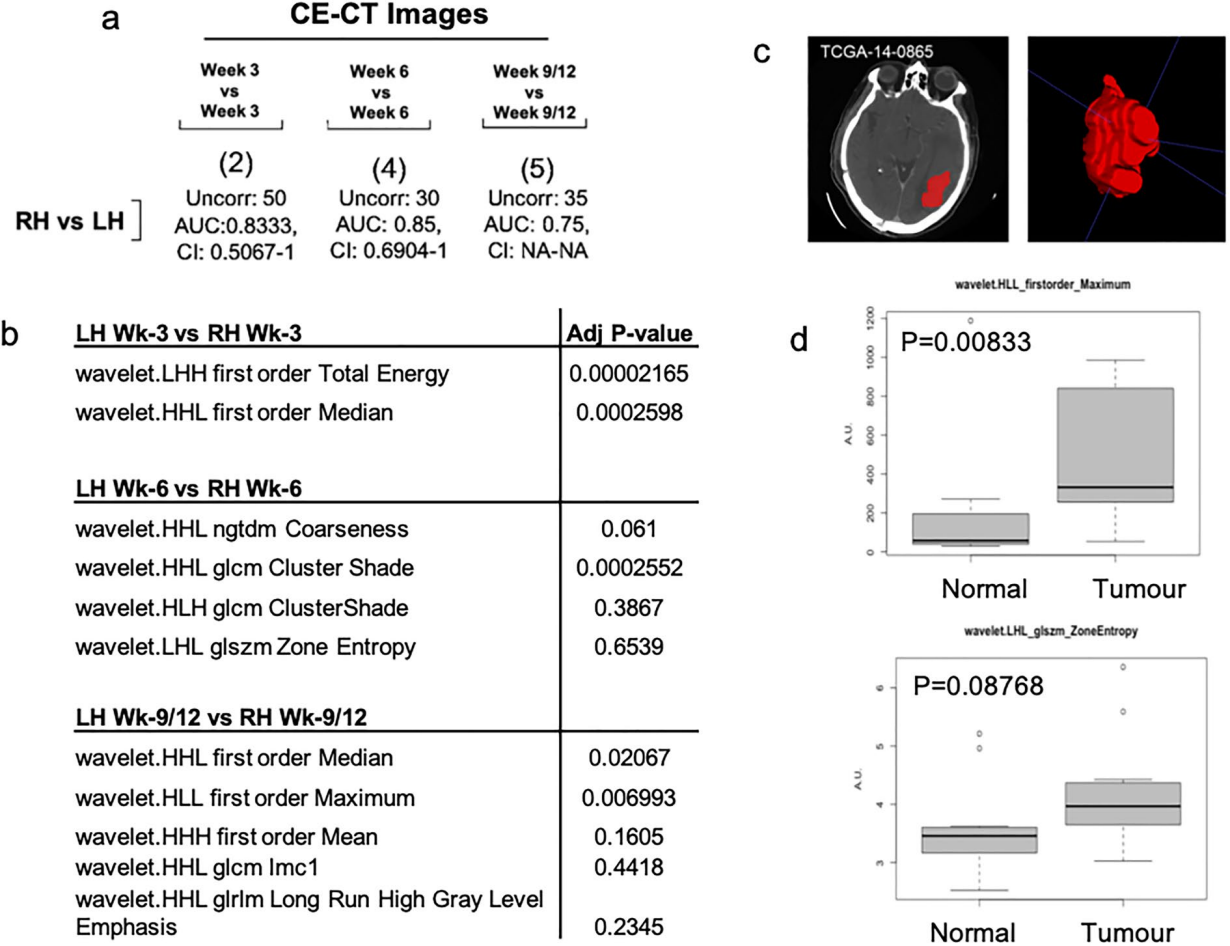
*Assessment of features altered between normal and tumour regions in preclinical and clinical images.* (**a**) Best Subset feature number (determined via RFE analysis) for each comparison is indicated within parentheses. Remaining uncorrelated feature number following pair-wise Spearman correlation, and AUC and ROC results are indicated for each comparison. (**b**) Tabular representation of Optimum features identified in inter-hemisphere comparisons (LH *vs* RH). (**c**) Representative coronal CE-CT slice of TCIA-TCGA GBM tumour delineation and generated 3D VOI (red). (**d**) Box plots of most significant features identified in total LH *vs* RH analysis analysed in tumour *vs* normal features. *P*-value < 0.05 deemed significant, Wilcoxon rank sum test.

We first confirmed that novel preclinical GBM CT-radiomic pipelines facilitate discrimination of tumour presence within the tumour-implanted hemisphere of the brain (RH). To this end we examined whether CT-radiomic features may assist in distinguishing between non-tumour-implanted (LH) and tumour-implanted (RH) rodent brain regions across each timepoint. Specifically, we assessed inter-hemisphere (LH *vs* RH) features at week-3, week-6 and week-9/12 (Fig. 4a). Comparison of LH and RH features via recursive feature analysis (RFE) (Fig. S3a–c), identified a panel of wavelet filtered first order, ngtdm, glcm, glszm and glrlm features which effectively differentiate between LH and RH (Fig. 4a, Fig. S3d–f). In all (Fig. 4a), significantly altered features (*n* = 2–5) were identified and performance in validation set generated an AUC > 0.7. By way of summary, Fig. 4b tabulates features identified as the best feature subset following RFE analysis. Taken together, CT-radiomic features may therefore assist in distinguishing between the tumour-implanted hemisphere and non-tumour-implanted hemisphere in orthotopic rodent GBM models. Furthermore, these tumour-region specific features may facilitate confirmation of tumour growth when BLI is unavailable.

### Performance of preclinical CT-radiomics features in patient datasets: proof of concept

To ascertain preclinical-clinical feature relevance and investigate mouse-to-human species conservation, pre-clinical, tumour-specific features were assessed in an exploratory, publicly available patient CE-CT cohort (*n* = 10) downloaded from ‘The Cancer imaging archive’ (TCIA) (Fig. 4c,d, S7). This cross-sectional cohort comprises a 1:2.3 ratio of female to male patients. Moreover 60% of patients were > 60 years of age upon diagnosis, with 30% of patients surviving > 1 year post diagnosis. IDH/MGMT status was unavailable for this cohort (supplementary Fig. S7). Features identified as significantly altered (*n* = 11 features) in the inter-hemisphere (LH/non-tumour-implanted hemisphere *vs *RH/tumour-implanted hemisphere) analyses were univariately analysed between normal and tumour regions of clinical GBM CT images. As summarised in Fig. S4, several features show altered distributions in tumour regions, with wavelet HLL first order maximum (*p* = 0.00833) significantly altered between normal and tumour regions. Moreover, wavelet LHL glszm zone entropy displayed increased intensity (*p* = 0.08768) in tumour regions (Fig. 4d). Markedly, analogous trends towards increased intensity in tumour *vs* normal clinical CT regions of these features tentatively suggests feature conservation across species.

### Evaluation of CT radiomic features for early tumour detection

**Figure 5:**
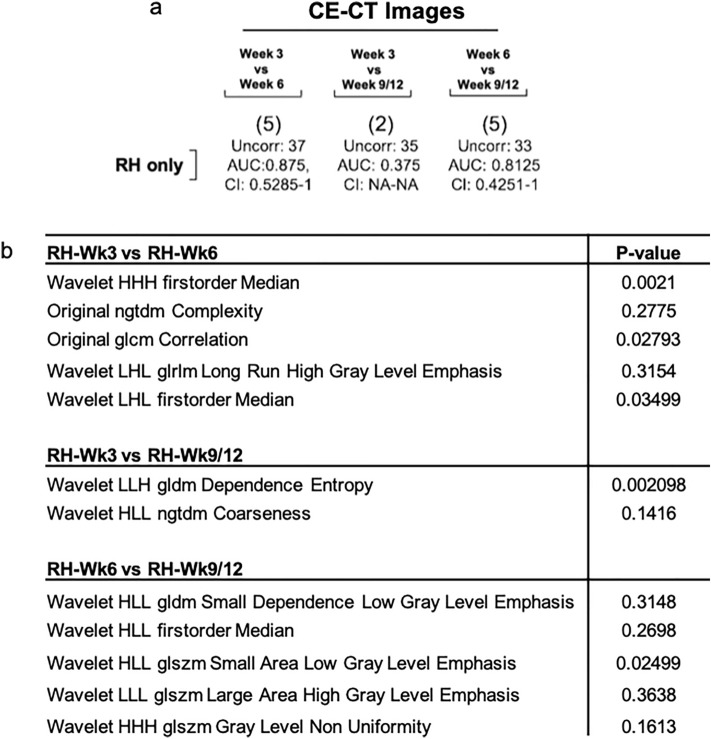
*Evaluation of CT radiomic features for early tumour detection.* A decision tree-based recursive feature elimination method was applied to the training dataset and RFE performed to select features of importance. Receiver operating characteristic (ROC) area under the curve (AUC) analysis following comparison of (**a**) CE-CT images across timepoints and (**b**) Tabular representation of features identified in timepoints comparisons. *P*-value < 0.05 deemed significant, Wilcoxon rank sum test.

**Figure 6:**
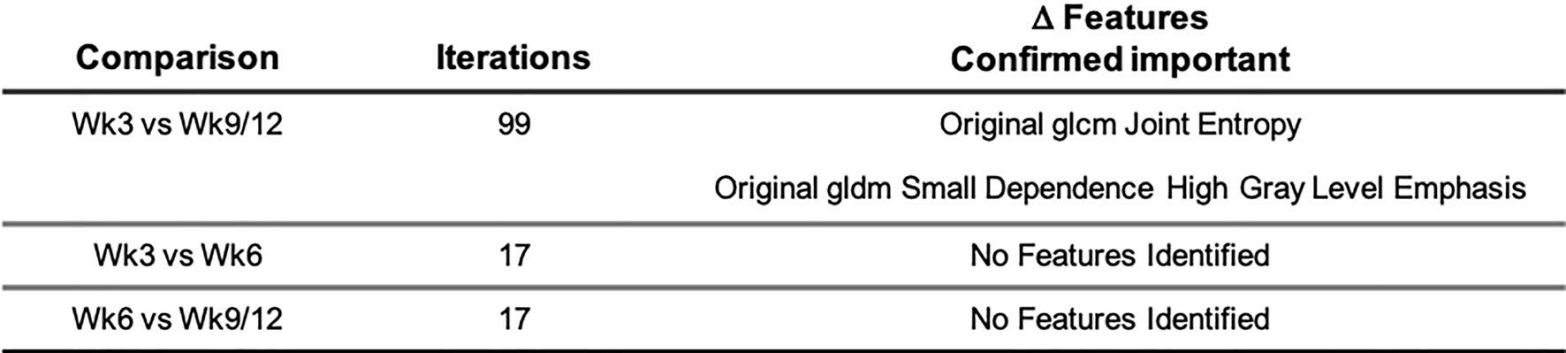
*Delta radiomic analysis of relative feature change across timepoints*. The Boruta algorithm was implemented to assess importance of features within DRF dataset. *P* < 0.05 deemed significant. Wilcoxon rank sum test.

We lastly sought to determine if CT-radiomic features could support tumour detection as early as experimental BLI (Week-1) or prior to tumour detection by visual assessment of CT images. To achieve this, we applied both conventional (Fig. 5) and delta radiomics (Fig. 6) approaches. Specifically we assessed radiomic features in our initial CT scan at week-3, where tumours were not visually detectable and compared to week-6 and week-9/12. A five-feature classifier was identified upon comparison of the tumour-implanted hemisphere (RH) early timepoint features at week-3 and week-6 (Performance in validation set; AUC: 0.86, 95% CI 0.53–1), which may be capable of distinguishing between early RH timepoints (Fig. S5a). Furthermore, comparison of mid (Week-6) and late (Week-9/12) features within the tumour-implanted hemisphere (RH) identifies a five-feature classifier (including both original and wavelet filtered features) with a performance in validation set of AUC: 0.81 (95% CI 0.43–1; Fig. 5b, S5c). In contrast, analysis of week-3 and week-9/12 features within the tumour region (RH) did not generate an effective classifier for differentiation between mid- and late timepoints (Performance in validation set; AUC: 0.38, 95% CI NA-NA). Here the ROC curve indicates inverted performance (Fig. S5b). By way of summary, Fig. 5b tabulates features identified as the best feature subset following RFE analysis (Fig. S5d–f). Taken together, these data demonstrate that radiomic features from the CE-CT scans may assist in earlier discrimination of the tumour-implanted hemisphere. Moreover, CT-radiomic features can identify the tumour-implanted hemisphere 3–6 weeks before tumours are detectable on visual inspection of images. (NB: longitudinal radiomic features were not verified in clinical datasets due to the absence of a longitudinal clinical imaging dataset).

### Evaluation of CT delta radiomic features for early tumour detection

We next evaluated whether analysis of net feature change between normal tissue (LH) and tumour region (RH) could improve the ability of CT-radiomic features to determine tumour presence. Here, the Boruta algorithm, where features compete with shadow attributes rather than among themselves, was applied (Fig. S6). Firstly, comparison of week-3 and week-9/12 delta radiomic features (DRFs) identified two features significantly altered between timepoints as ‘confirmed important’ (Z-score of feature > Maximum Z-score among shadow attributes). This analysis identified original glcm Joint Entropy as significantly increased (*p* = 0.001954) and original gldm small dependence high gray level emphasis as significantly decreased (*p* = 0.03301) in week-9/12 compared to week-3 (Fig S6a,b). Comparison of week-3 and week-6 DRFs, and week-6 and week-9/12 DRFs, rejected all other features as potential classifiers (Figs. 6, S6a). Figure 6 illustrates DRFs identified following timepoint comparison. Overall, these data indicate that normalisation to the corresponding hemisphere in which there is no tumour presence, and therefore delta radiomics, may indeed improve the utility of CT-radiomic features for early tumour detection in certain contexts. As above, features now require verification in longitudinal, clinical patient datasets.

## Discussion

In the current study we have established preclinical CT-radiomic workflows and have shown that CT-radiomic features have the potential to distinguish the tumour-implanted hemisphere from the non-tumour-implanted hemisphere, thereby supporting early detection of tumours during longitudinal analysis. Moreover in a proof of concept study we have successfully translated preclinical radiomic pipelines to patient datasets. In this context, pre-clinical CT-features interrogated in clinical datasets demonstrated analogous trends in intensities (i.e. similarly increased intensity in regions of tumour tissue), suggesting species conservation^30^.

Firstly, we employed the invasive^24^, orthotopic U87R-Luc2 GBM model (xenograft) to optimise preclinical brain tumour radiomic pipelines and identify radiomic features which may indicate tumour presence. Initially, CE-CT images were visually assessed, with only 16% of tumours visually discernible on average (Fig. S1d). We next interrogated CE-CT images to optimise radiomic feature analysis and assess if radiomic features could assist in discrimination of the tumour-implanted hemisphere compared to the non- tumour-implanted hemisphere. Strict reduction of redundant features (removal of shape and highly inter-correlated features) and implementation of robust statistical methods were employed throughout this study. Moreover, open-source software and tools were implemented to ensure reproducibility of the preclinical pipeline. When radiomic features in tumour and normal tissue were compared, we identified a three-feature classifier which putatively discriminates between the non-tumour-implanted hemisphere (LH) and tumour-implanted hemisphere (RH) regions. Aligning with previous studies, here LLH firstorder total energy wavelet feature was identified. Total Energy is the value of Energy feature, scaled by the voxel volume (cubic mm)^25^, and has previously been identified as significantly altered between Grade II and III gliomas (MRI)^26^. Similarly, GLCM cluster shade (measure of skewness and uniformity) which is significantly increased (representing greater asymmetry about the mean) in tumour regions in our analyses has been utilised in the discrimination of high grade and lower grade primary brain tumours^27^. Therefore, these features may collectively facilitate discrimination of tumour-implanted and non-tumour implanted hemispheres. Further validation in larger cohorts is now required.

Next, features identified in preclinical CE-CTs were assessed in an exploratory, clinical TCGA-TCIA cohort of newly diagnosed GBMs^28^. As no robust longitudinal clinical datasets were available for analysis, radiomic features identified as discriminators of normal *vs* tumour tissue, were univariately assessed for alterations between normal and tumour regions. Analogous trends in First Order Maximum (indicative of the maximum gray level) and Zone Entropy feature intensity (a higher zone entropy value indicates more texture heterogeneity) across animal and patient CTs were identified, suggesting species conservation. Moreover, entropy has been previously shown to correlate with glioma grade^27^, further indicating features identified in the preclinic may indeed translate to the clinic.

Lastly, our results indicate that CT-radiomic features could support earlier detection of tumour presence when features were compared between timepoints (early RH (tumour-implanted hemisphere)-timepoint (Week-3 *vs* Week-6) comparison, and RH-mid (Week-6) and RH-late (Week-9/12)). Here we identified a number of wavelet gray level emphasis features (a higher value indicates a greater concentration of high gray-level values present) which have been shown to have prognostic utility when considered alongside GBM clinical predictors^29^. Moreover, identified among these features, NGTDM Coarseness (A measure of average difference between the center voxel and its neighbourhood—higher values indicates a more uniform texture) has been shown to be significantly altered in the early thermographic detection of breast cancer^30^. Overall, these data suggest that comparison of pre-clinical CT-radiomic features across timepoints represents a viable strategy for identification of imaging biomarkers for early brain tumour detection. Indeed, interrogation and verification of these features in longitudinal datasets which incorporate clinical characteristics such as IDH mutation and MGMT methylation status, patient age and overall survival as additional variables is now warranted. Notably, raw radiomic features values vary in intensity between datasets due to inter-scanner differences and key differences between clinical and pre-clinical scanners (E.g. scan energies, voxel size etc.). Therefore future expanded preclinical-clinical studies, which multivariately assess CT-radiomic features, will require harmonisation of data (E.g. via the ComBAT method).

To refine and further exploit the availability of longitudinal data, relative net change in preclinical GBM CT-radiomic features was also studied^31^. It has previously been shown that Delta radiomic features (DRF; ∆) may generate an improved performance compared to standard radiomics signatures^32^. DRF analysis was therefore implemented to interrogate DRF ability to facilitate early tumour prediction. Interrogation of net relative differences between LH and RH identified a number of features of importance. Among the features identified by the Boruta algorithm, GLCM Joint entropy (randomness/variability in neighbourhood intensity values) has previously been identified as indicative of oesophageal tumour stage^33^. Overall, the identification of these features indicates that CT-features representative of heterogeneity may be a suitable marker for tumour presence in preclinical orthotopic GBM CE-CTs.

While small animal CT imaging is not without challenges, including diffuse tissue margins and relatively poor soft tissue contrast^34,35^, we have established a clinically relevant CT-radiomics pipeline for application in rodent brain tumour models. Notably, while the RANO criteria have not yet been translated to the preclinical setting, the clinical Response Evaluation Criteria in Solid Tumours (RECIST) criteria^23^ has been adapted for use in PDX clinical trials (mRECIST)^24,25^, indicating the strong value in the preclinical translation of clinical workflows, and ultimately improving translatability of preclinical models. Indeed, application of both conventional and delta CT-radiomics may enhance the utility of CT when applied to rodent brain tumour models, which may thus represent a clinically relevant and viable alternative to invasive biological sampling. Importantly, randomisation of outcomes throughout these analyses resulted in undefined AUC’s and CIs, indicating that despite relatively low cohort animal numbers in certain cases, the established feature analysis pipeline produced rational predictions.

Overall, limitations of this study include relative low sample numbers in both preclinical (experimental group numbers limited due to study design and adherence to the 3Rs) and clinical cohorts (lack of availability of public, longitudinal GBM CE-CT datasets). Future studies will require test–retest analyses to ensure robustness and reliability of features over time. In addition, studies which implement an expanded panel of GBM models, directly comparing tumour size (via harvested tissue and IHC), tumour infiltration pattern (to account for tumour growth within the needle tract) and radiomic features, are warranted. Moreover, these studies would benefit from comparative, quantitative MRI to assist in overcoming discrepancies arising in the estimation of tumour burden and tumour progression when BLI is used alone, and when tumours are located close to the skull^36,37^. These studies will underpin the future application of specific, tumour-region CT-radiomic features in earlier tumour detection. Therefore, to build on this hypothesis generating work, additional studies are now warranted. These studies should implement more frequent, longitudinal CT imaging, more adequately powered preclinical imaging groups and comparative MR imaging. Likewise, studies of radiomic features in a panel of GBM PDXs could yield classifiers which are progressively more clinically translatable compared to xenograft GBM models^38^. Finally, development of deep learning methods to remove labour intensive and oftentimes subjective tumour segmentation is required.

In conclusion, this study demonstrates a promising role for pre-clinical CT-radiomic pipelines. Our data supports the application of a pre-clinical CT-radiomics strategy as an alternative monitoring method for rodent brain tumour models when BL and MR imaging is unavailable. Notably, we illustrate that identified radiomic features have capacity for translation to clinical images, indicating that preclinical models may provide discovery platform for clinically relevant radiomic features. Overall, we demonstrate the potential for preclinical CT-radiomic classifiers to support the detection of orthotopic brain tumours which are otherwise difficult to detect visually.

## Methods

### Cell culture

The invasive^24,39^ human GBM cell line U87R-Luc2 (gift from Peter Forsyth, Moffitt Cancer Center, Tampa, FL) was cultured in Dulbecco’s Modified Eagle’s Medium (DMEM) F12 supplemented with 10% fetal bovine serum and 400 mg/mL G418^22^.

### GBM orthoxenograft studies

In vivo studies were licensed and approved by the Heath Products Regulatory Authority (License number AE18982/P123) and University College Dublin’s Animal Research Ethics Committee (Protocol number P17-21). Animal experiments were carried out under Directive 2010/63/EU of the European Parliament on protection of animals used for scientific purposes. All sections of this manuscript adhere to ARRIVE Guidelines.

In vivo studies, which adhere to the PREPARE guidelines, were performed at the University College Dublin specific pathogen free (SPF) facility. Mice imported from Charles River UK (Cambridge, UK) were housed in groups of 4–6 in individually ventilated cages (Techniplast, London, UK) and given access to food and water ad libitum. A 12 h light/12 h dark cycle, 40–50% humidity and temperature of 18–22 °C were maintained throughout. Environmental enrichment of red polycarbonate houses, pura wooden chew stick, nesting material and clear plastic tunnels were provided. Animals were visually assessed daily to monitor overall health status. Experimental animal cohort numbers were calculated based on Workman et al.^40^.

For tumour implantations, NOD/SCID (4–6 weeks; female; 18–22 g) were anesthetized with O2/isoflurane mixture (1.5% isoflurane in 100% O2) and fixed in a stereotaxic frame. 2 × 10^5^ of U87R-Luc2 cells (NOD/SCID [*n* = 20]) were orthotopically implanted at a depth of 2 mm into the right hemisphere (RH) as previously described^22^. Adverse effects were scored on a multi-category scale and a rodent coma scale monitored neurological status.

In vivo data were analysed using GraphPad Prism (GraphPad Software, San Diego, CA USA) or Excel (Microsoft, Redmond, WA, USA). Student’s t-test, and ANOVA were performed to assess statistical significance. Statistical analysis of in vivo data was performed using a log-rank test for survival studies with *p* < 0.05 deemed significant.

### In vivo study design

A cohort of U87R-Luc2 tumour-bearing mice (n = 10) underwent repeated CE-CT imaging at week-3, week-6, week-9 and week-12 post implantation (Fig. 2a). Late timepoint (Week-9 and Week-12) images were combined for analyses due to low number of remaining animals (*n* = 3 and *n* = 2 remaining at Week-4).

*Bioluminescence imaging:* Weekly BLI was performed^22^ with an IVIS Spectrum (Perkin Elmer, Waltham, MA, USA) as previously described. Imaging was carried out under anesthesia (1.5% isoflurane/100% O_2_) on a heated stage. 15 min prior to imaging, mice received 150 mg/kg luciferin (Perkin Elmer, Waltham, MA, USA) via subcutaneous injection. A 1 s reference image was taken (binning = 4, F-stop = 1). Living Image software (V4.3.1, Perkin Elmer Waltham, MA, USA) was utilised for BLI image analysis and average radiance (p/s/cm2/sr) was analysed.

### Immunohistochemistry

Post-mortem, whole brains were excised, rinsed in Dulbecco’s-(D)PBS solution and fixed in 4% formaldehyde (48 h). Tissues were embedded in paraffin and 5 μm thick sections cut. Routine hematoxylin and eosin (H&E) staining was performed to facilitate histological evaluation. Standard immunohistochemistry methods were used to stain for Ki67 and CD-31 (Mouse IgG; 1:150 dilution; Cell Signaling Technology, London, UK).

### Preclinical CT Image acquisition and reorientation

Longitudinal CT images were acquired on the TRIUMPH^X-O-CT^ system (LabPet 4, X-O CT) (1.5% isoflurane/100% O_2_). 0–1 min prior to imaging 300ul warmed iodine based-contrast (300 mg iodine/mL^41^) was administered via intravenous lateral tail-vein injection. CE-CT images were acquired at 50 kV, 200 projections, voxel size 120 µm, FOV 61.44 mm, slice thickness 2 mm and 88 µA. Delineation of each reconstructed CT DICOM was carried out using PMOD (version 3.9; PMOD Technologies). DICOMs were reoriented to the same focal plane for visual assessment (Fig. S1). To prevent inter-observer variability, two experts semi-automatically delineated regions of interest (ROI). 3D Volume of interests (VOIs) were delineated for both right hemisphere (RH; tumour-implanted hemisphere) and the left hemisphere (LH; non-tumour-implanted hemisphere), with a threshold determined per image to exclude bone from the generated bounding boxes. Specifically, features that completely represent the tumour-implanted hemisphere were selected via segmentation of the right hemisphere (RH). Features that represent the non-tumour-implanted hemisphere were selected via segmentation of the left hemisphere (LH).

### Patient CT Image acquisition and segmentation

Cross-sectional and publicly available GBM patient CT (*n* = 10) images were downloaded from The Cancer Genome Atlas Glioblastoma Multiforme (TCGA-GBM) data collection^42^ (The Cancer imaging archive (TCIA)^28^ (https://www.cancerimagingarchive.net/)). Tumour and normal tissue were manually delineated from baseline (pre-treatment) scans (Fig. 4e) using the polygon tool and thresholding in ITKSnap software^34^.

### CT-radiomic feature extraction

Radiomic features are extracted over the whole ROI representing the left or right hemisphere using PyRadiomics (V2.7.7) (Harvard Medical School, Boston, MA, USA)^25^. Extraction parameters applied were: original = {}, wavelet = {}, bin width = 25, correctMask = true, interpolator = 2, resampledPixelSpacing = [2.5, 0.390625, 0.390625] (‘Extraction Parameter File’, Supplementary Materials). A fixed bin width of 25 was used for discretisation of the image grey level. Feature classes extracted (*n* = 843 total features) include First Order Statistics, Shape-based features, Gray Level Cooccurence Matrix (GLCM), Gray Level Run Length Matrix (GLRLM), Gray Level Size Zone Matrix (GLSZM), Neighbouring Gray Tone Difference Matrix (NGTDM), Gray Level Dependence Matrix (GLRLM), and finally the same features extracted from wavelet filtered versions of images. Mathematical definitions of each feature is located in online pyRadiomics documentation.

### Analytical pipeline

R software (version 3.5.2, R Foundation for Statistical Computing, Vienna, Austria; http://www.r-project.org) was employed for all the statistical analysis.

*Reduction of preclinical data dimensionality:* To avoid overfitting (large number of extracted features (*n* = 843) and low sample number can find many spurious correlations), all shape features were removed (*n* = 14, Fig. S2; highly conserved between LH and RH). Remaining features underwent predictive analysis^38^ as follows. In all, outcome was assigned (0-LH; Normal tissue, 1-RH; tumour tissue) and datasets partitioned into training and validation cohort (80%:20% random split). Inter-correlated features were removed (determined in training dataset by pairwise Spearman correlation (> 0.85^43^) and removal of most highly correlated features). *Reduction of clinical data dimensionality:* All shape features (*n* = 14) were removed from downstream analyses.

*Analysis of preclinical data:* A decision tree-based recursive feature elimination method was applied to the training dataset, and recursive feature elimination performed using the recursive feature elimination (RFE) function (Caret package, version 6.0–84^41^; Cross-Validated (CV) via Repeated CV, five-fold, repeated 5 times). Most informative and non-redundant CT-radiomic features were selected to train a random forest, and to measure performance area under the receiver operator characteristic (ROC) curve (AUC) was calculated (pROC version 1.15.3). AUC, sensitivity and specificity are reported for each RCV (Fig. 3b). R packages implemented throughout are tabulated within supplementary materials (Supplementary Table 1). Assignment of randomised outcomes and re-analysis via the aforementioned pipeline was subsequently performed to validate detection of signal *vs* noise (Data not shown). *Analysis of clinical data:* CT-radiomic features identified in the analyses of preclinical data (LH/non- tumour-implanted hemisphere *vs* RH/tumour-implanted hemisphere analysis) were univariately analysed across normal and tumour regions of segmented clinical GBM CT images. Trends in feature intensity between tumour and normal regions were identified and demonstrated via box plot. Longitudinal radiomic features were not verified in clinical datasets due to the absence of a longitudinal clinical imaging dataset.

*Delta Radiomic analysis:* Feature-level changes were calculated by the relative net change between features from LH and RH (∆_*features*_ = *[features*_*RH*_*-features*_*LH*_*]/features*_*LH*_). The Boruta algorithm^44^ (version 7.0.0), which implements a wrapper around a random forest classification algorithm, was employed to identify delta radiomic features of importance (Fig. S6) across timepoints (Week-3, -6, -9/12). Importance scores were generated across features and Tentative Rough Fix performed to assess weaker features. Features ‘confirmed important’ exhibit a feature Z-score > Maximum Z-score among shadow attributes.

### Ethics approval and consent to participate

Protocols were approved by the University College Dublin Animal Research Ethics Committee. Animal experiments were carried out under Directive 2010/63/EU of the European Parliament on protection of animals used for scientific purposes. All sections of this manuscript adhere to ARRIVE Guidelines. All methods were carried out in accordance with relevant guidelines and regulations.

### Supplementary Information

Supplementary Information.

## Data Availability

The datasets analysed during this study are available from the corresponding author on reasonable request.

## Abbreviations

95% CI: 95% Confidence interval
AUC: Area under the curve
BLI: Bioluminescence imaging
CRC: Colorectal cancer
CT: Computed tomography
GBM: Glioblastoma
MRI: Magnetic resonance imaging
OS: Overall survival
VOI: Volume-of-interest
