# Supplementary material for: A clinically relevant computed tomography (CT) radiomics strategy for intracranial rodent brain tumour monitoring
Source: Sci Rep. 2024 Feb 1;14:2720. doi: 10.1038/s41598-024-52960-1 (PMC10834979; doi:10.1038/s41598-024-52960-1)
Supplement: Supplementary file 1 — Supplementary Information. [file 41598_2024_52960_MOESM1_ESM.pptx]

## Slide 1
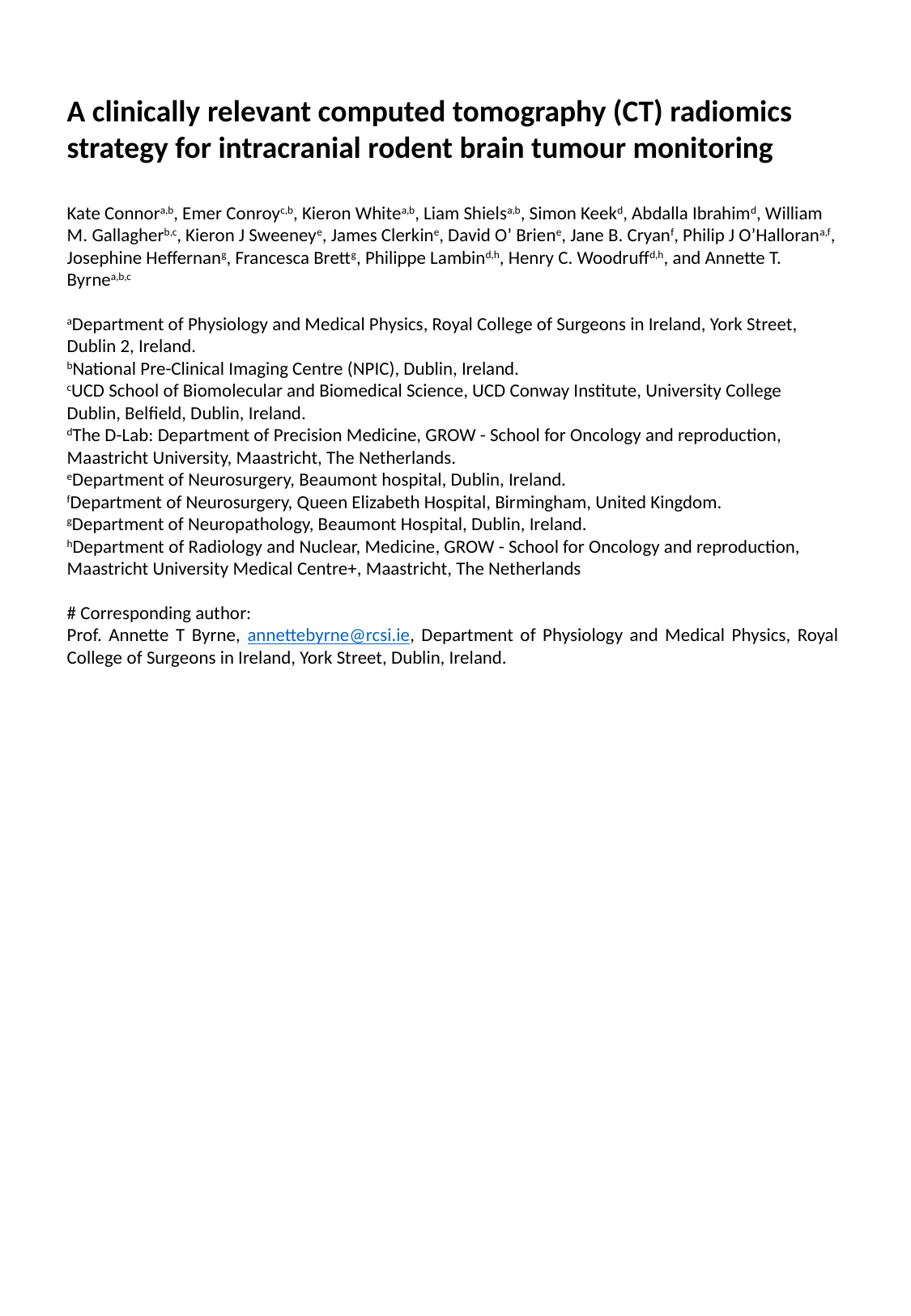

A clinically relevant computed tomography (CT) radiomics strategy for intracranial rodent brain tumour monitoring
Kate Connora,b, Emer Conroyc,b, Kieron Whitea,b, Liam Shielsa,b, Simon Keekd, Abdalla Ibrahimd, William M. Gallagherb,c, Kieron J Sweeneye, James Clerkine, David O’ Briene, Jane B. Cryanf, Philip J O’Hallorana,f, Josephine Heffernang, Francesca Brettg, Philippe Lambind,h, Henry C. Woodruffd,h, and Annette T. Byrnea,b,c
aDepartment of Physiology and Medical Physics, Royal College of Surgeons in Ireland, York Street, Dublin 2, Ireland.
bNational Pre-Clinical Imaging Centre (NPIC), Dublin, Ireland.
cUCD School of Biomolecular and Biomedical Science, UCD Conway Institute, University College Dublin, Belfield, Dublin, Ireland.
dThe D-Lab: Department of Precision Medicine, GROW - School for Oncology and reproduction, Maastricht University, Maastricht, The Netherlands.
eDepartment of Neurosurgery, Beaumont hospital, Dublin, Ireland.
fDepartment of Neurosurgery, Queen Elizabeth Hospital, Birmingham, United Kingdom.
gDepartment of Neuropathology, Beaumont Hospital, Dublin, Ireland.
hDepartment of Radiology and Nuclear, Medicine, GROW - School for Oncology and reproduction, Maastricht University Medical Centre+, Maastricht, The Netherlands
# Corresponding author:
Prof. Annette T Byrne, annettebyrne@rcsi.ie, Department of Physiology and Medical Physics, Royal College of Surgeons in Ireland, York Street, Dublin, Ireland.

## Slide 2
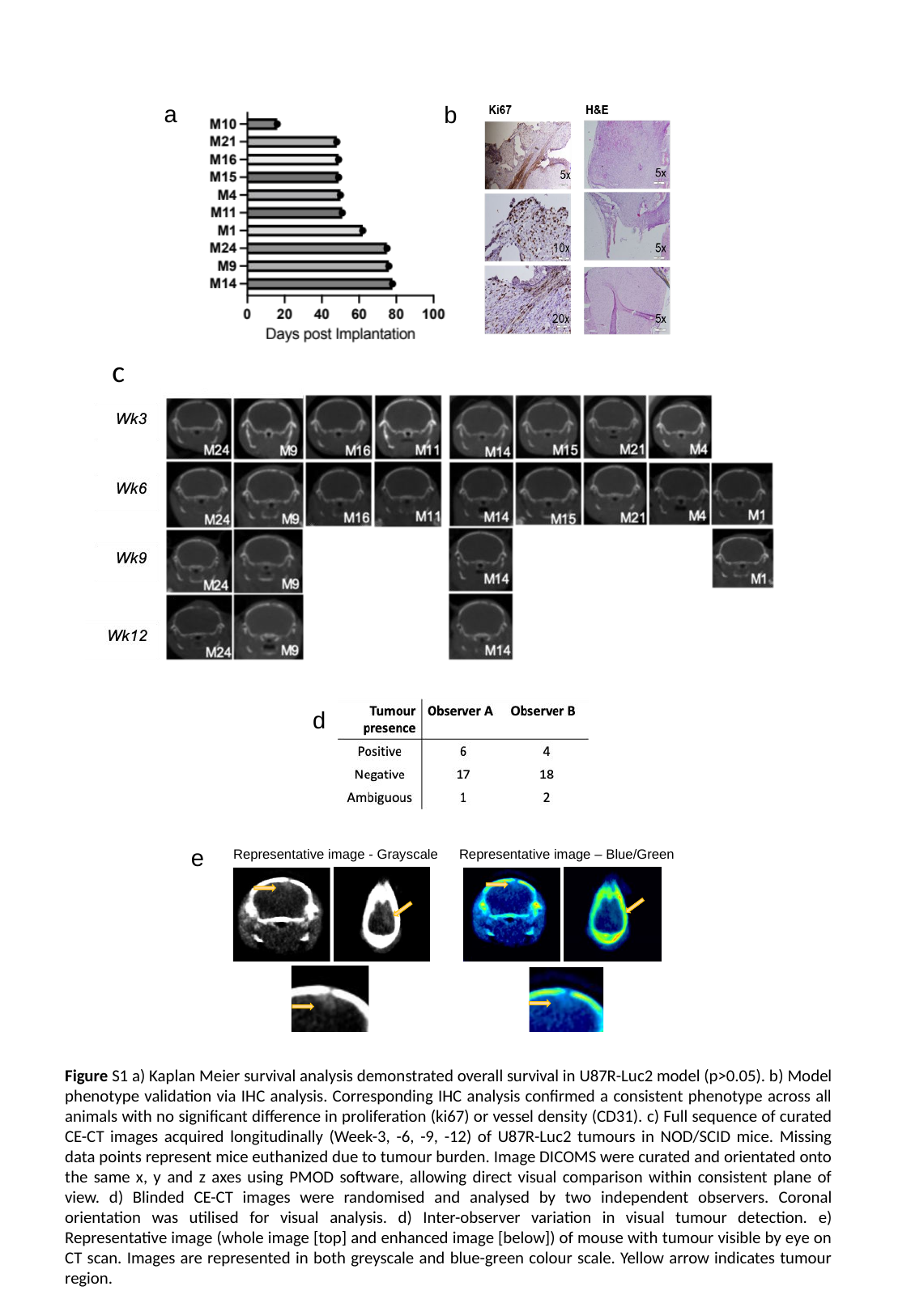

a
b
c
d
e
Representative image – Blue/Green
Representative image - Grayscale
Figure S1 a) Kaplan Meier survival analysis demonstrated overall survival in U87R-Luc2 model (p>0.05). b) Model phenotype validation via IHC analysis. Corresponding IHC analysis confirmed a consistent phenotype across all animals with no significant difference in proliferation (ki67) or vessel density (CD31). c) Full sequence of curated CE-CT images acquired longitudinally (Week-3, -6, -9, -12) of U87R-Luc2 tumours in NOD/SCID mice. Missing data points represent mice euthanized due to tumour burden. Image DICOMS were curated and orientated onto the same x, y and z axes using PMOD software, allowing direct visual comparison within consistent plane of view. d) Blinded CE-CT images were randomised and analysed by two independent observers. Coronal orientation was utilised for visual analysis. d) Inter-observer variation in visual tumour detection. e) Representative image (whole image [top] and enhanced image [below]) of mouse with tumour visible by eye on CT scan. Images are represented in both greyscale and blue-green colour scale. Yellow arrow indicates tumour region.

## Slide 3
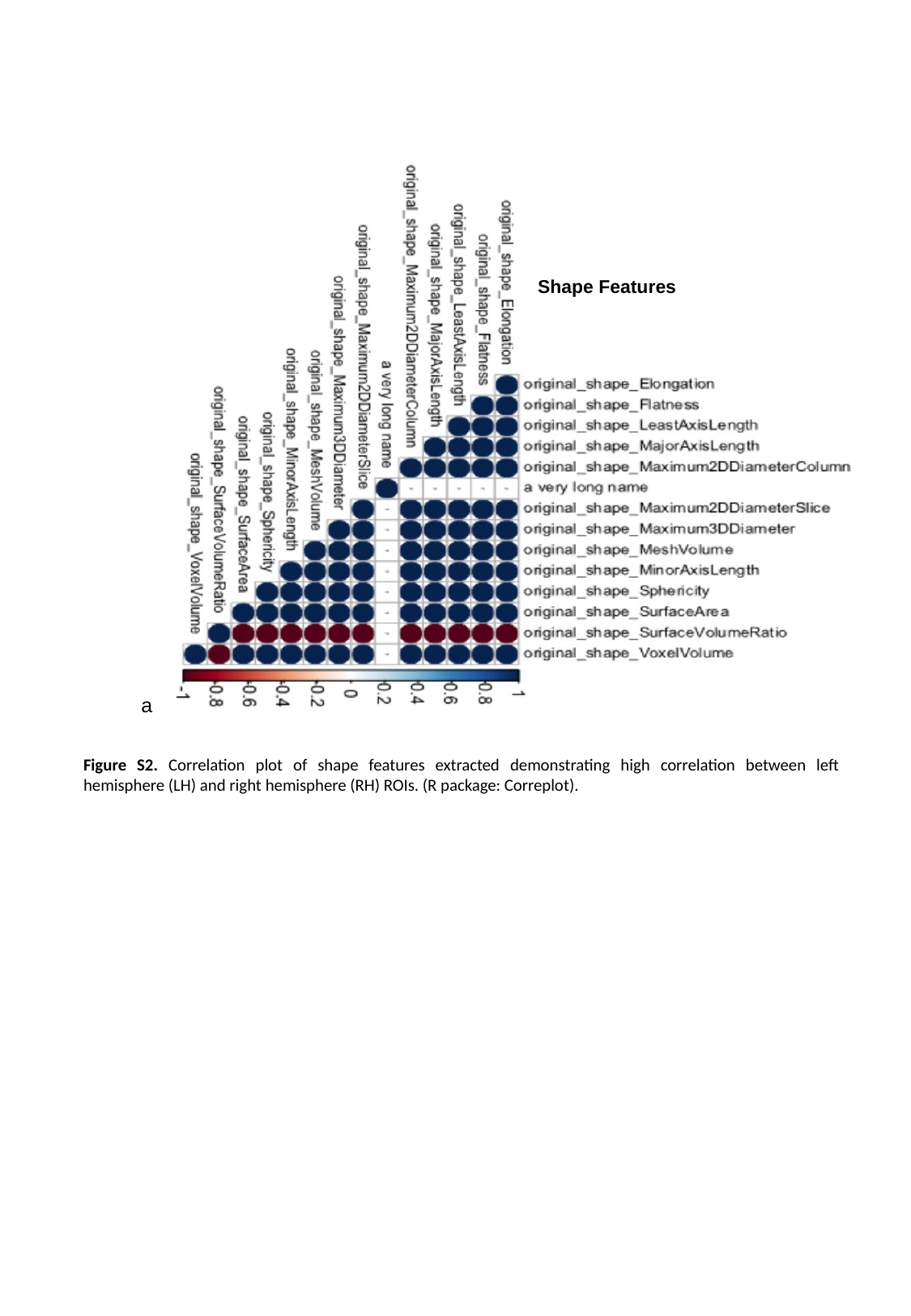

Shape Features
a
Figure S2. Correlation plot of shape features extracted demonstrating high correlation between left hemisphere (LH) and right hemisphere (RH) ROIs. (R package: Correplot).

## Slide 4
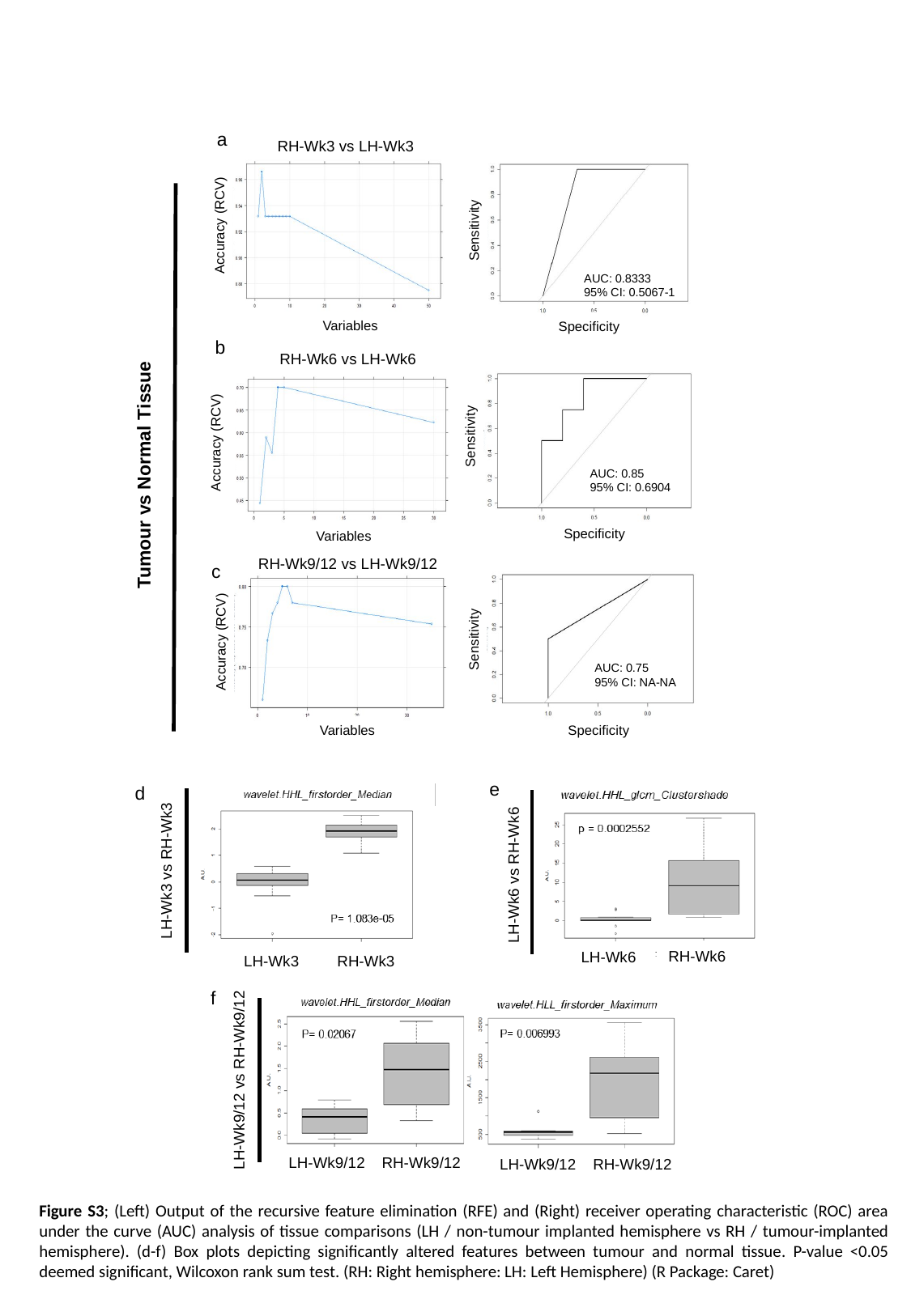

a
RH-Wk3 vs LH-Wk3
Accuracy (RCV)
Sensitivity
AUC: 0.8333
95% CI: 0.5067-1
Variables
Specificity
b
RH-Wk6 vs LH-Wk6
Sensitivity
Accuracy (RCV)
AUC: 0.85
95% CI: 0.6904
Specificity
Variables
Tumour vs Normal Tissue
RH-Wk9/12 vs LH-Wk9/12
Sensitivity
Accuracy (RCV)
AUC: 0.75
95% CI: NA-NA
Variables
Specificity
c
e
LH-Wk6 vs RH-Wk6
RH-Wk6
LH-Wk6
d
LH-Wk3 vs RH-Wk3
LH-Wk3
RH-Wk3
f
LH-Wk9/12 vs RH-Wk9/12
LH-Wk9/12
RH-Wk9/12
LH-Wk9/12
RH-Wk9/12
Figure S3; (Left) Output of the recursive feature elimination (RFE) and (Right) receiver operating characteristic (ROC) area under the curve (AUC) analysis of tissue comparisons (LH / non-tumour implanted hemisphere vs RH / tumour-implanted hemisphere). (d-f) Box plots depicting significantly altered features between tumour and normal tissue. P-value <0.05 deemed significant, Wilcoxon rank sum test. (RH: Right hemisphere: LH: Left Hemisphere) (R Package: Caret)

## Slide 5
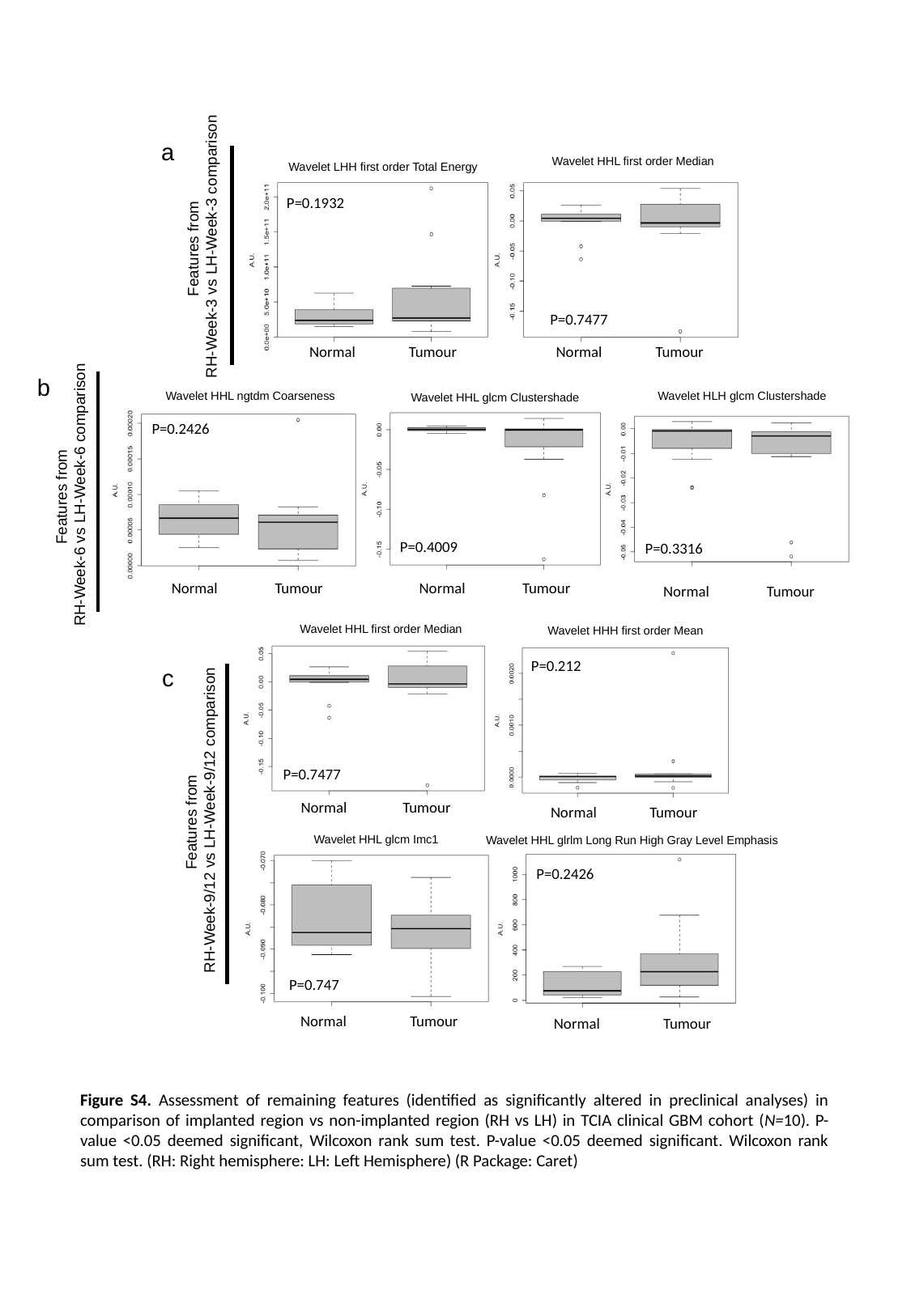

Features from
 RH-Week-3 vs LH-Week-3 comparison
P=0.1932
P=0.7477
Normal
Tumour
Normal
Tumour
Features from
RH-Week-6 vs LH-Week-6 comparison
P=0.4009
Normal
Tumour
P=0.2426
Normal
Tumour
P=0.3316
Normal
Tumour
P=0.7477
Normal
Tumour
P=0.212
Tumour
Normal
P=0.2426
Tumour
Normal
P=0.747
Tumour
Normal
a
b
c
Features from
RH-Week-9/12 vs LH-Week-9/12 comparison
Wavelet HHL first order Median
Wavelet LHH first order Total Energy
Wavelet HLH glcm Clustershade
Wavelet HHL ngtdm Coarseness
Wavelet HHL glcm Clustershade
Wavelet HHL first order Median
Wavelet HHH first order Mean
Wavelet HHL glcm Imc1
Wavelet HHL glrlm Long Run High Gray Level Emphasis
Figure S4. Assessment of remaining features (identified as significantly altered in preclinical analyses) in comparison of implanted region vs non-implanted region (RH vs LH) in TCIA clinical GBM cohort (N=10). P-value <0.05 deemed significant, Wilcoxon rank sum test. P-value <0.05 deemed significant. Wilcoxon rank sum test. (RH: Right hemisphere: LH: Left Hemisphere) (R Package: Caret)

## Slide 6
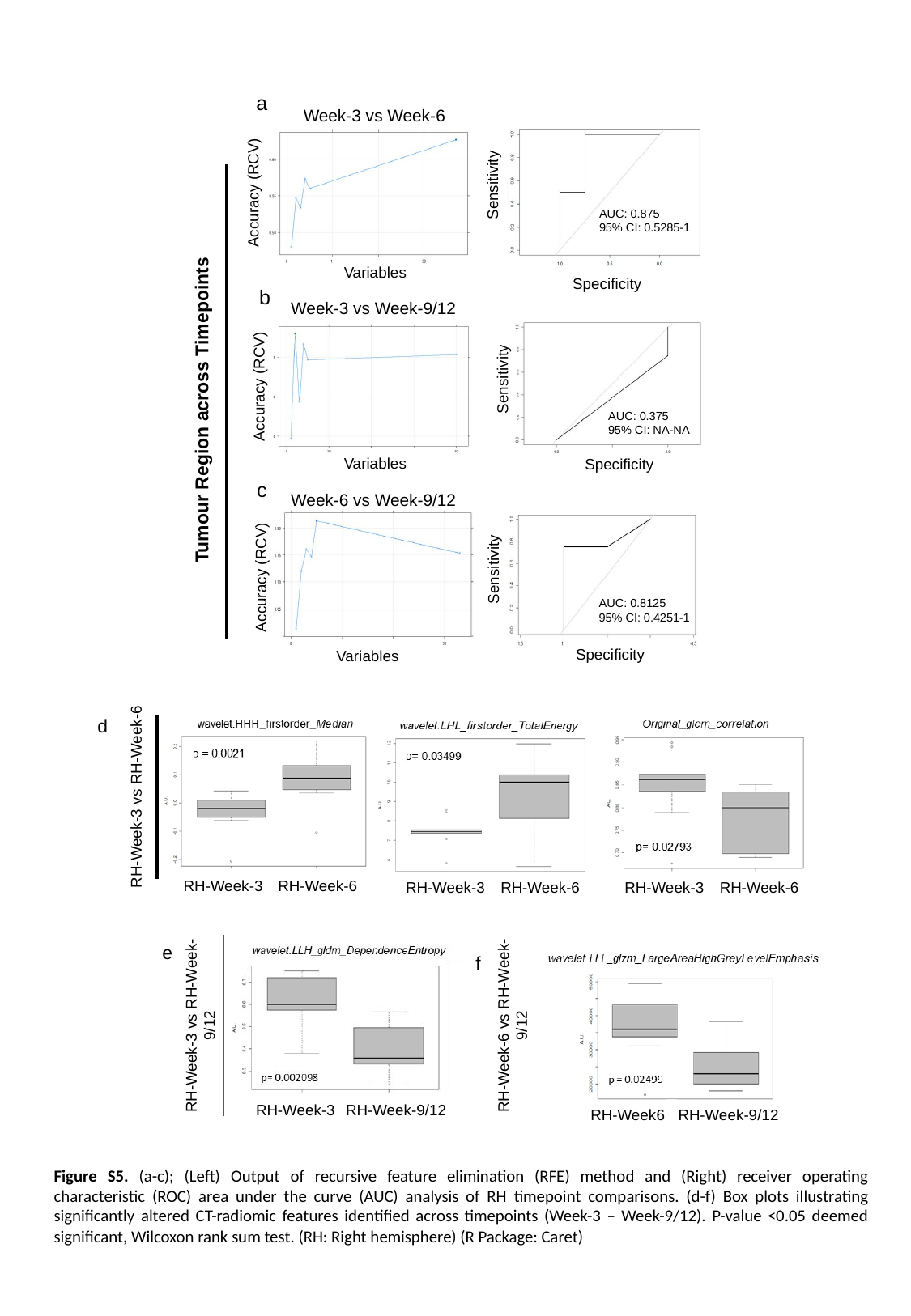

a
Week-3 vs Week-6
Sensitivity
Accuracy (RCV)
AUC: 0.875
95% CI: 0.5285-1
Variables
Specificity
b
Week-3 vs Week-9/12
AUC: 0.375
95% CI: NA-NA
Sensitivity
Accuracy (RCV)
Tumour Region across Timepoints
Variables
Specificity
c
Week-6 vs Week-9/12
Sensitivity
Accuracy (RCV)
AUC: 0.8125
95% CI: 0.4251-1
Specificity
Variables
d
RH-Week-3 vs RH-Week-6
RH-Week-3
RH-Week-6
RH-Week-3
RH-Week-6
RH-Week-3
RH-Week-6
e
RH-Week-3 vs RH-Week-9/12
f
RH-Week-6 vs RH-Week-9/12
RH-Week-3
RH-Week-9/12
RH-Week6
RH-Week-9/12
Figure S5. (a-c); (Left) Output of recursive feature elimination (RFE) method and (Right) receiver operating characteristic (ROC) area under the curve (AUC) analysis of RH timepoint comparisons. (d-f) Box plots illustrating significantly altered CT-radiomic features identified across timepoints (Week-3 – Week-9/12). P-value <0.05 deemed significant, Wilcoxon rank sum test. (RH: Right hemisphere) (R Package: Caret)

## Slide 7
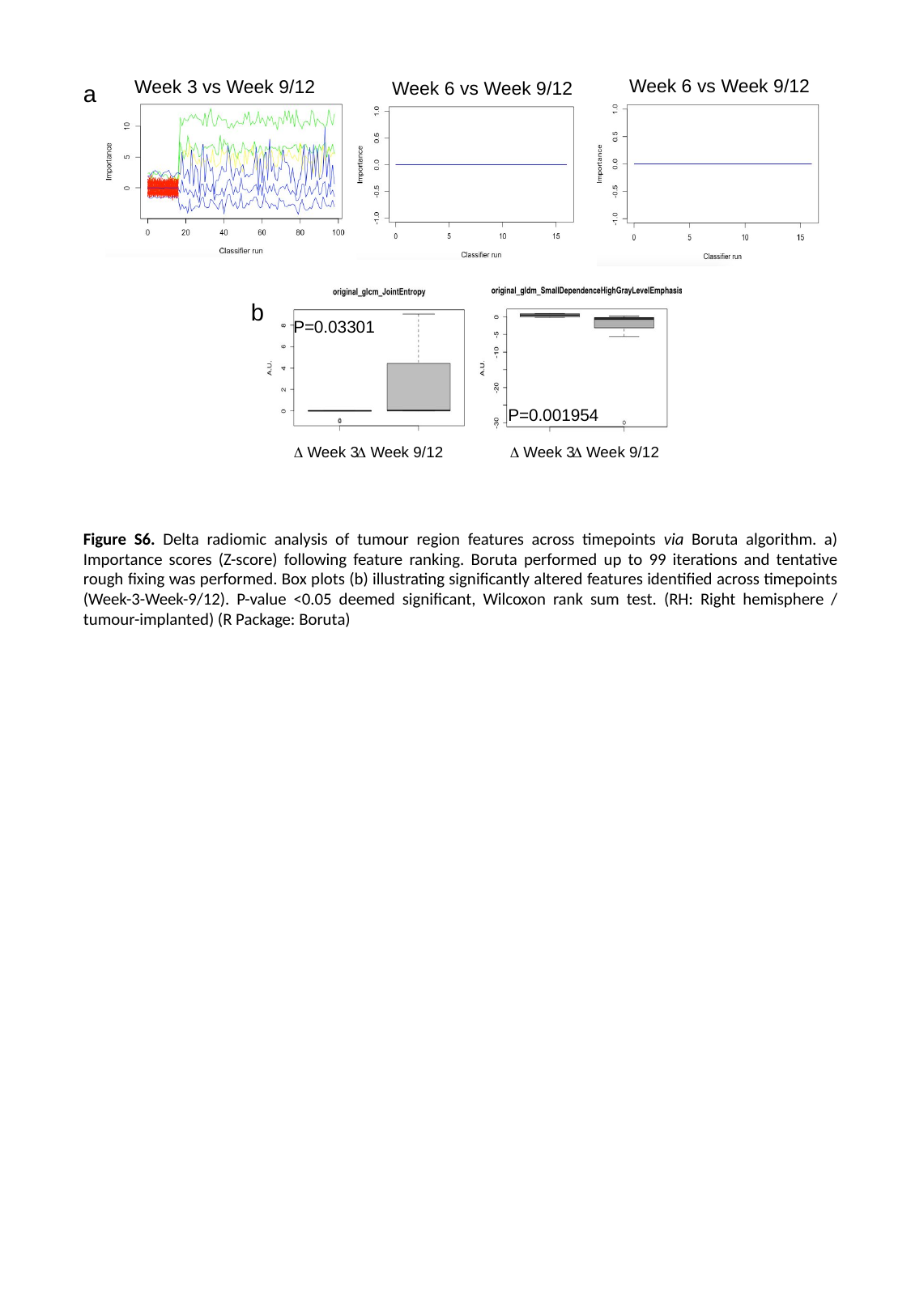

Week 6 vs Week 9/12
Week 3 vs Week 9/12
a
Week 6 vs Week 9/12
P=0.001954
D Week 9/12
D Week 3
D Week 9/12
D Week 3
P=0.03301
b
Figure S6. Delta radiomic analysis of tumour region features across timepoints via Boruta algorithm. a) Importance scores (Z-score) following feature ranking. Boruta performed up to 99 iterations and tentative rough fixing was performed. Box plots (b) illustrating significantly altered features identified across timepoints (Week-3-Week-9/12). P-value <0.05 deemed significant, Wilcoxon rank sum test. (RH: Right hemisphere / tumour-implanted) (R Package: Boruta)

## Slide 8
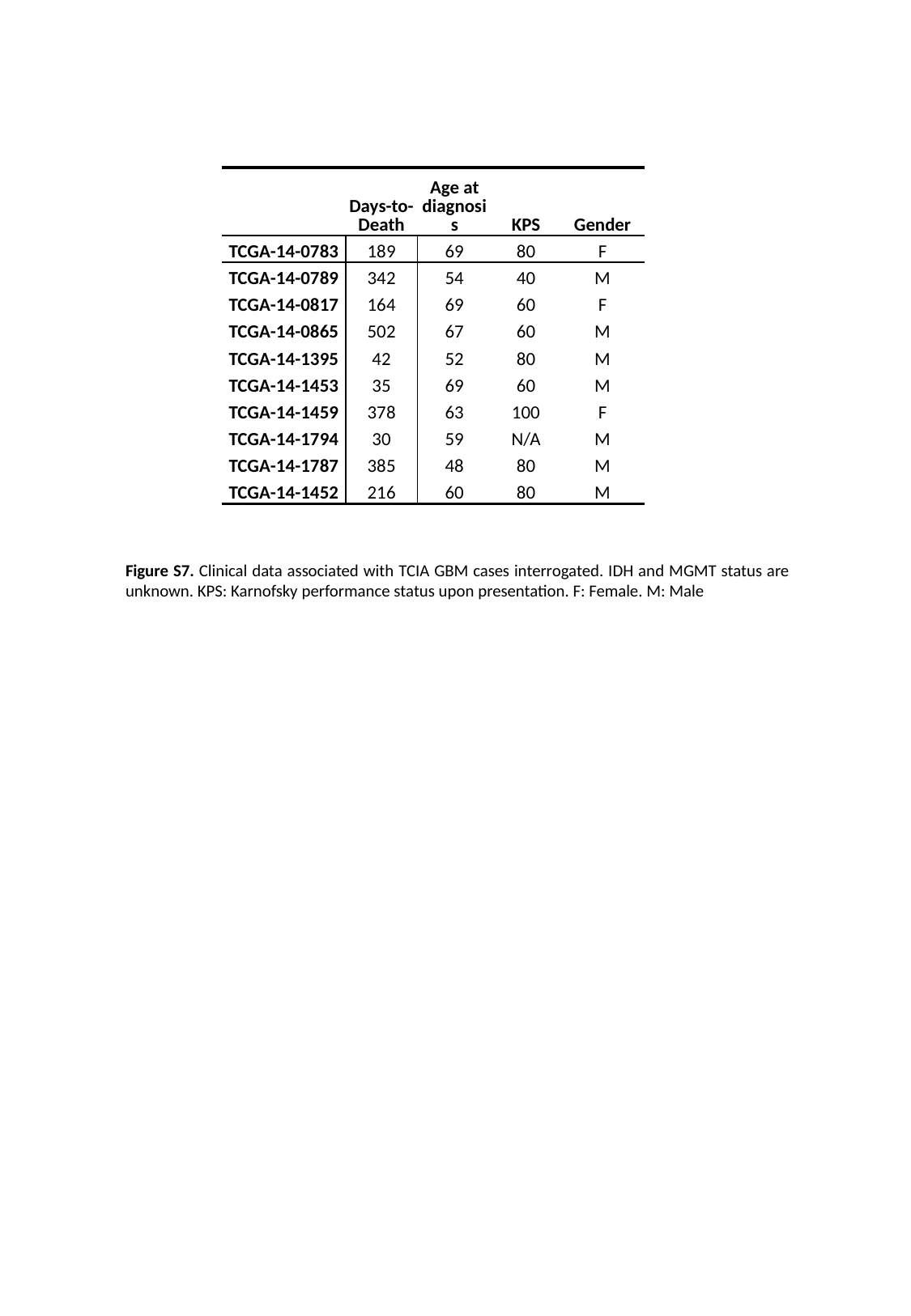

| | Days-to-Death | Age at diagnosis | KPS | Gender |
| --- | --- | --- | --- | --- |
| TCGA-14-0783 | 189 | 69 | 80 | F |
| TCGA-14-0789 | 342 | 54 | 40 | M |
| TCGA-14-0817 | 164 | 69 | 60 | F |
| TCGA-14-0865 | 502 | 67 | 60 | M |
| TCGA-14-1395 | 42 | 52 | 80 | M |
| TCGA-14-1453 | 35 | 69 | 60 | M |
| TCGA-14-1459 | 378 | 63 | 100 | F |
| TCGA-14-1794 | 30 | 59 | N/A | M |
| TCGA-14-1787 | 385 | 48 | 80 | M |
| TCGA-14-1452 | 216 | 60 | 80 | M |
Figure S7. Clinical data associated with TCIA GBM cases interrogated. IDH and MGMT status are unknown. KPS: Karnofsky performance status upon presentation. F: Female. M: Male

## Slide 9
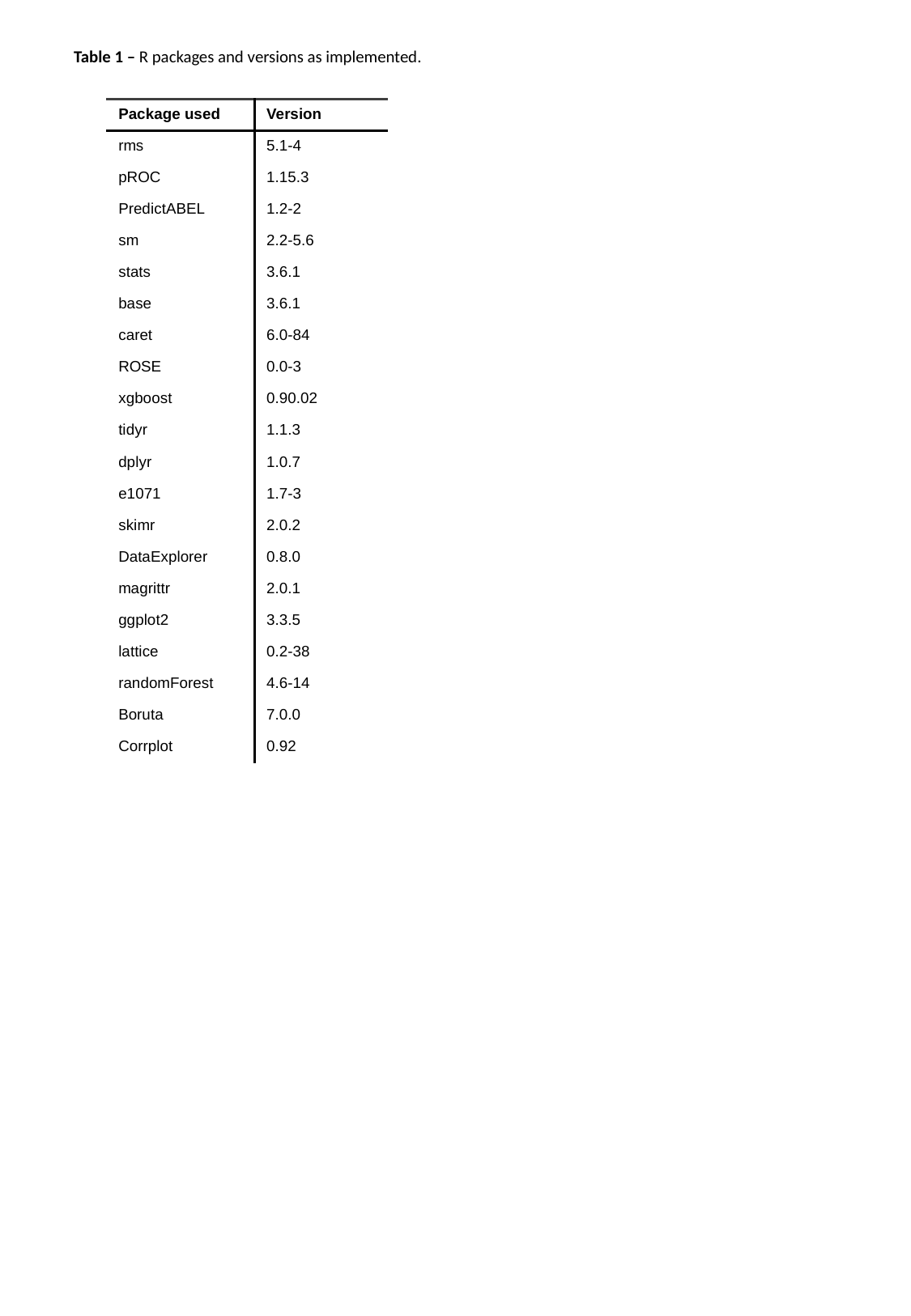

Table 1 – R packages and versions as implemented.
| Package used | Version |
| --- | --- |
| rms | 5.1-4 |
| pROC | 1.15.3 |
| PredictABEL | 1.2-2 |
| sm | 2.2-5.6 |
| stats | 3.6.1 |
| base | 3.6.1 |
| caret | 6.0-84 |
| ROSE | 0.0-3 |
| xgboost | 0.90.02 |
| tidyr | 1.1.3 |
| dplyr | 1.0.7 |
| e1071 | 1.7-3 |
| skimr | 2.0.2 |
| DataExplorer | 0.8.0 |
| magrittr | 2.0.1 |
| ggplot2 | 3.3.5 |
| lattice | 0.2-38 |
| randomForest | 4.6-14 |
| Boruta | 7.0.0 |
| Corrplot | 0.92 |
